# Self-Generated Gradients Yield Exceptionally Robust Steering Cues

**DOI:** 10.3389/fcell.2020.00133

**Published:** 2020-03-05

**Authors:** Luke Tweedy, Robert H. Insall

**Affiliations:** Cancer Research UK Beatson Institute, Glasgow, United Kingdom

**Keywords:** chemotaxis, neutrophils, *Dictyostelium*, 3D migration, mitogens, receptors

## Abstract

Chemotaxis is a widespread mechanism that allows migrating cells to steer to where they are needed. Attractant gradients may be imposed by external sources, or self-generated, when cells create their own steep local gradients by breaking down a prevalent, broadly distributed attractant. Here we show that chemotaxis works far more robustly toward self-generated gradients. Cells can only respond efficiently to a restricted range of attractant concentrations; if attractants are too dilute, their gradients are too shallow for cells to sense, but if they are too high, all receptors become saturated and cells cannot perceive spatial differences. Self-generated gradients are robust because cells maintain the attractant at optimal concentrations. A wave can recruit varying numbers of steered cells, and cells can take time to break down attractant before starting to migrate. Self-generated gradients can therefore operate over a greater range of attractant concentrations, larger distances, and longer times than imposed gradients. The robustness is further enhanced at low cell numbers if attractants also act as mitogens, and at high attractant concentrations if the enzymes that break down attractants are themselves induced by constant attractant levels.

## Introduction

Chemotaxis is cell migration directed by gradients of diffusible chemicals (Insall and Andrew, 2007). It is seen throughout biology, particularly in development where chemotaxis steers processes such as germ cell migration (Renault and Lehmann, 2006), melanoblast spreading (Tabone-Eglinger et al., 2012) and neural connection (Lewellis et al., 2013). It is also vital to immune function, where leukocytes use chemotaxis to locate tissue damage, infections, and one another.

Most publications describe a simplistic view of chemotaxis, in which cells respond to attractant gradients that have been set up by other biological entities [a “source”, which creates the chemoattractant, and a “sink”, which removes it; the gradient runs predictably from source to sink (Zigmond, 1977; Insall, 2013)]. In this narrative, a gradient is imposed by others, and responding cells are passive, doing nothing to change the gradient. In most cases, the imposed gradient (Tweedy et al., 2016b) model is far too simple to be accurate. Cells often adjust the gradient with dedicated enzymes or by endocytosing the attractant together with its receptors. Despite this, imposed gradients remain the lens through which most chemotaxis data are viewed.

An opposite extreme is fully “self-generated” gradients, in which the chemoattractant is initially homogeneous, with no particular source or sink. Instead, populations of cells work together to break down attractant, meaning regions with high cell density soon have very low attractant levels. Diffusion between these low-concentration regions, and nearby areas with no cells and thus high attractant levels, creates attractant gradients (Tweedy et al., 2016b). In this case the cells respond actively, changing the gradient as they migrate up it, and always chasing a retreating zone of high attractant concentration. One reason such systems are often ignored is that they are very hard to measure or predict in detail. They are made dynamically, and controlled by both positive and negative feedback as cells migrate toward attractants while breaking them down.

In most biological scenarios there are elements of both imposed and self-generated gradients. Attractants are rarely produced truly homogeneously; but in almost all scenarios where it has been studied, cells endocytose or degrade chemoattractants while responding to them. This article examines whether self-generation adds robustness to chemotactic responses as well as shaping the gradients.

In both types of gradient, imposed and self-generated, there are two key points to bear in mind. First, all the cells’ information comes through saturable cell-surface receptors, so the binding properties of the receptors are key to understanding chemotaxis. Second, it is the gradient, rather than the presence of an attractant, that determines chemotaxis. A high but homogeneous level of chemoattractant yields the same outcome as none at all – there is no chemotactic information, so cells are not steered (their motility may be altered, a separate process known as “chemokinesis,” but the direction of chemokinesis is random). Together, these points mean that chemotaxis is only efficient at attractant concentrations near the dissociation constant (K*_*d*_*) of the receptors. At lower concentrations too few attractant molecules are detected; at higher attractant concentrations the receptors become saturated, so the cell cannot measure the difference between the attractant levels at its front and rear. This means that imposed chemotactic gradients are only efficient if attractant concentration is within an order of magnitude either side of the K*_*d*_*. They can also only work over relatively short distances, because the steepness of an imposed gradient must be spread over the entire path (Tweedy et al., 2016a). Self-generated gradients involve cells changing the attractant concentrations around themselves. They therefore have different constraints, which allow them to run over greater distances, and whose effect on robustness is explored in this article.

There are many different ways that cells can establish local gradients. In the current work we only consider gradients established by cell breaking down broadly distributed attractants. Cell typically use two different methods to drop the local concentrations – they can endocytose receptors when they are bound to ligands (Donà et al., 2013), thereby removing the ligand for proteolysis or chemical breakdown, or they can use cell-surface enzymes like MMPs (McQuibban et al., 2002), lipid phosphatases (Susanto et al., 2017) and phosphodiesterases (Garcia et al., 2009). The endocytosis pathway can be reinforced by specific “scavenger” receptors, which remove ligand without necessarily transducing a signal, as occurs when the zebrafish lateral line primodium migrates (Donà et al., 2013; Venkiteswaran et al., 2013). Under the conditions we are studying (high concentrations of attractant, significant local volumes) the enzymes dominate so most of the work we describe considers enzymes, but there is no theoretical difference between the two – each removes attractant at a characteristic rate and with an optimal local concentration. Most chemotactic cells, indeed, use both mechanisms simultaneously; but as our previously published model of *Dictyostelium* chemotaxis demonstrates (Tweedy et al., 2016a), simply considering cell-surface enzymes is sufficient to predict cell behavior with a high degree of accuracy, so we only consider this mechanism here.

## Results

### Chemotaxis to Self-Generated Gradients Is Robust to Attractant Concentration

Many widely used chemotaxis assays use improbably high ligand concentrations (e.g., Laevsky and Knecht, 2001; Sapey et al., 2014), orders of magnitude greater than the measured affinities of the receptors. This is strong evidence that cells in these assays shape their gradients by locally breaking down the attractants, even if they are initially presented from one direction. This implies that cells can modulate gradients to suit themselves, which could allow more robust chemotaxis. Interestingly, such high concentrations are often physiologically appropriate – serum may contain micromolar LPA, even though the receptors’ K*_*d*_*s are in the low nanomolar range (Susanto et al., 2017).

**FIGURE 1:**
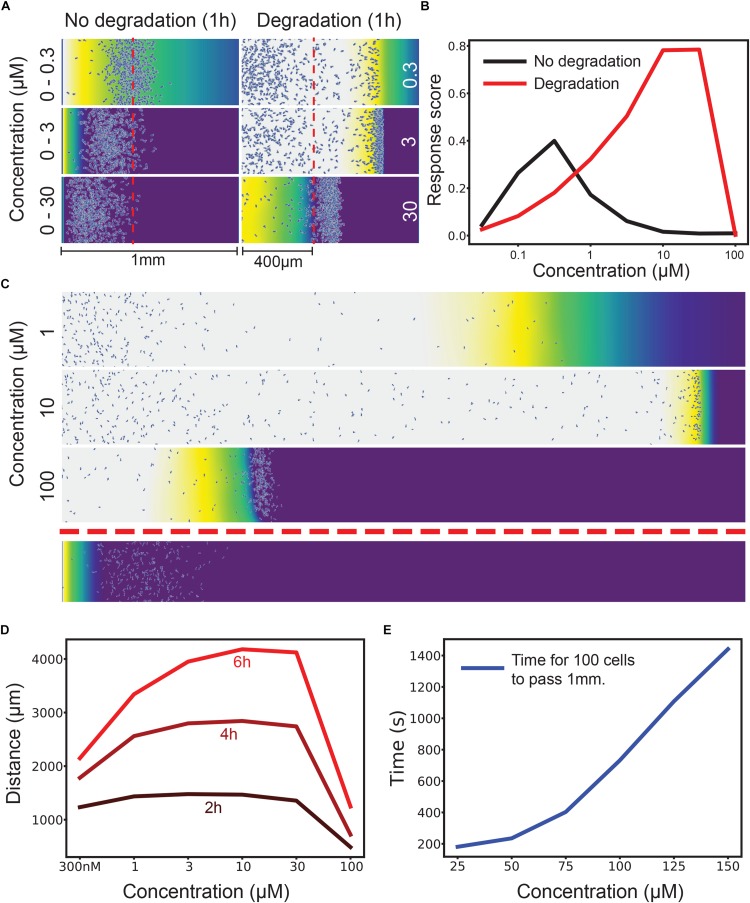
Attractant degradation increases the range and concentration robustness of chemotaxis. **(A)** Simulations of a gradient chamber assay with and without attractant degradation after 1 h at low (0–300 nM gradient vs uniform 300 nM), intermediate (0–3 μM vs uniform 3 μM), and high concentrations (0–30 μM vs uniform 30 μM). **(B)** Fraction of cells (response score) passing 400 μm after 1 h across a range of concentrations with (red) and without (black) attractant degradation. **(C)** Example long range chemotaxis of self-generated gradient across three orders of magnitude. At 1 μM, the gradient produced by cells eventually becomes too shallow for optimal chemotaxis. At 10 μM, the gradient remains steep and the wave of cells remains cohesive. At 100 μM, the cells are at first unable to degrade attractant as rapidly as diffusion delivers it to them, causing a delay before the leading wave of cells forms. Though this wave performs well once formed, it covers less distance over the allotted time due to this delay effect. Shown below is the response to a linear gradient of 0–10 μM over the same distance. **(D)** Distance covered by the leading 10 cells at 2, 4, and 6 h by a self-generated gradient at various concentrations. **(E)** Time taken for 100 cells to pass 1 mm across many saturating concentrations. The graph has distinct linear and non-linear regions, showing that, after a certain point, twice the attractant leads to (approximately) twice the delay before a wave develops.

To test the robustness to different concentrations we used an agent-based simulation that has been found to predict cell behavior accurately (Tweedy et al., 2016a). We ran two simulations of cells migrating across a 1 mm bridge between two large medium reservoirs, scoring cells as positive for chemotaxis if they migrated more than 400 μm in 1 h. In one, cells respond to a non-degradable attractant in an imposed gradient. In the other, cells respond to an initially homogenous stimulus that cells degrade locally to make a self-generated gradient. Figure 1A shows three different concentrations of attractant – with an imposed 0–300 nM gradient cells chemotax effectively, but at 0–3 μM few and 0–30 μM essentially no cells chemotax, because their receptors are so quickly saturated (we assume an attractant-receptor K*_*d*_* of 12 nM, which is appropriate for many naturally occurring small molecule attractants; Soede et al., 1994).

The self-generated gradient gives qualitatively different responses at each concentration – but in each case, cells chemotax efficiently. In 30 nM attractant a wave containing a few cells chemotaxes rapidly; in doing so it consumes all the chemoattractant, so many cells are left behind. At 300 nM a much denser wave is formed, including many of the cells; the wave moves at essentially the same speed. At 3 μM attractant the wave is denser still, and moves more slowly, because the cells’ ability to break down attractant is limited, and so they need time to break down enough attractant to create a local gradient.

Quantitative assessment (Figure 1B) of the proportion of cells that pass 400 μm along the track confirm this result. Imposed gradients have a peak around the K*_*d*_* of the receptor, then rapidly drop in efficiency; self-generated gradients increase in effectiveness as the attractant concentration gets higher and more cells are recruited to the front wave. The dynamic range of the self-generated gradients is clearly far higher, three orders of magnitude in this case. Counterintuitively, attractant degradation makes chemotaxis far more robust.

Of course, there are many ways of quantitating two such different assays; in this case, we have shown the imposed gradient to be more efficient than the self-generated one at low concentrations, because so few cells enter the leading wave; however, these few cells progress faster and more accurately, because the local gradient they form is so steep (Figure 1A, top right).

#### Contrasting Reasons for Failure at High and Low Attractant

Longer distances exaggerate the advantages of self-generated gradients. A 6 mm imposed gradient (Figure 1C, bottom) is essentially illegible at all attractant concentrations. This is generally true, as shown in our previous work (Tweedy et al., 2016a). Self-generated gradients are effective at a wide range of attractant concentrations, with higher concentrations becoming more efficient later in the assay (Figure 1D).

In the imposed gradient series, the ultimate cause of failure is the same for both the high- and low-concentration gradients (Figure 1B); there are only poor occupancy differences across the cell length. The reasons for this differ in the two cases, however. At low concentrations, the absolute gradient steepness is too shallow to create a proper leading signal. At high concentrations, almost all receptors are occupied a short distance up the gradient, meaning that very little occupancy difference can occur. The pattern is similar in self-generated gradients – at low concentrations, the steepest gradient the cell can create is limited in terms of receptor occupancy, and so cells do not maintain a perfect guidance cue for long (Figure 1C, 1 μM). At high concentrations, the flux of attractant by diffusion is able to match the maximal degradation rate that the cell population can achieve, leading to receptor saturation and reducing response (Figure 1C, 100 μM).

### Time Delay as a Source of Robustness

However, this offers another mechanism for robustness during self-generated gradient chemotaxis. If the source of attractant is finite, the cells can after a delay bring down high concentrations. Initially they see no gradient, but as they progressively lower the attractant concentration they eventually start chemotaxis. After this, their performance will closely match that of the maximal response, but with a time offset. Figure 1E shows the time for 100 cells to chemotax further than 1 mm in an assay like that in Figure 1C. The delay increases non-linearly at first, indicating the concentrations range where cells degrade below their maximum potential (from about 10× the reaction K*_*m*_*). Later increases are linear, limited only by the maximal degradation rate of the population of cells.

### Loss of Robustness at Low Cell Densities

**FIGURE 2:**
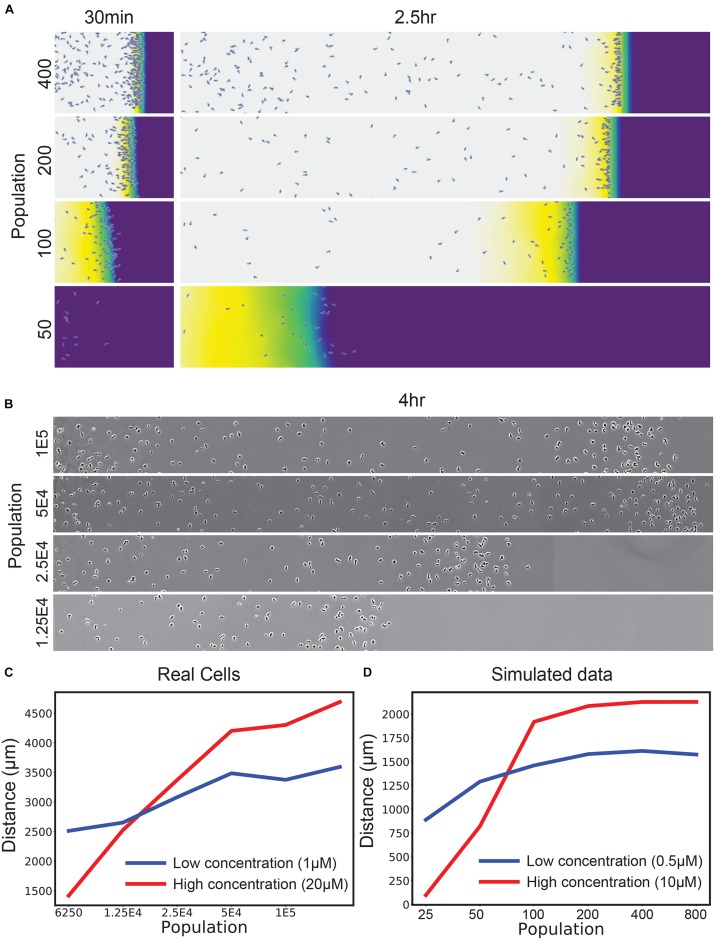
Self-generated gradients are robust to population changes unless saturated with attractant. **(A)** Simulations of the uniform 10 μM condition, varying the number of cells introduced at the left-hand side. **(B)** *D. discoideum* responding to an initially uniform profile of 20 μM folate, varying the initial number of cells in the assay in twofold steps. **(C)** Distance traveled by real *D. discoideum* cells migrating toward a 20 μM (red) or 1 μM (blue) background of the degradable attractant folate. *t*-test of means across the front-most cells × 3 biological repeats show significant differences in position (*p* < 0.01) for both the lowest and highest two populations. **(D)** Simulations of a range of populations for a 10 μM (red) and 500 nM (blue) attractant background.

This failure of self-generated chemotaxis at high attractant concentrations illustrates one aspect of self-generated gradients that is not robust. This is when too few cells are present. Figure 2A illustrates the decrease in chemotactic efficiency that occurs as the number of available cells drops (note the key parameter is the total V_*max*_ – the number of cells multiplied by the V_*max*_ for each cell; this will become important later). As the number of cells drops, at first the local gradient becomes less sharp, so chemotaxis is less efficient. At some point, however, the number of cells becomes too low to metabolize the attractant flux, causing a catastrophic drop in efficiency. We confirmed this simulated result with *D*ictyostelium *discoideum* cells migrating toward the degradable attractant folate under agarose (Laevsky and Knecht, 2001; Figure 2B). When assaying “normal” attractant levels (20 μM relative to a receptor K*_*d*_* of ∼12 nM), there is a simple fall-off of chemotaxis efficiency as the number of cells drops. We confirmed that this was due to an excess of attractant over cells by dropping the attractant levels to 1 μM (Figure 2C) – although peak chemotaxis decreased, as expected, the performance of small numbers of cells was enhanced. This is in line with the simulation prediction across a similar range of concentrations (Figure 2D).

#### 2D and 3D Chemotaxis Add a Level of Complexity

**FIGURE 3:**
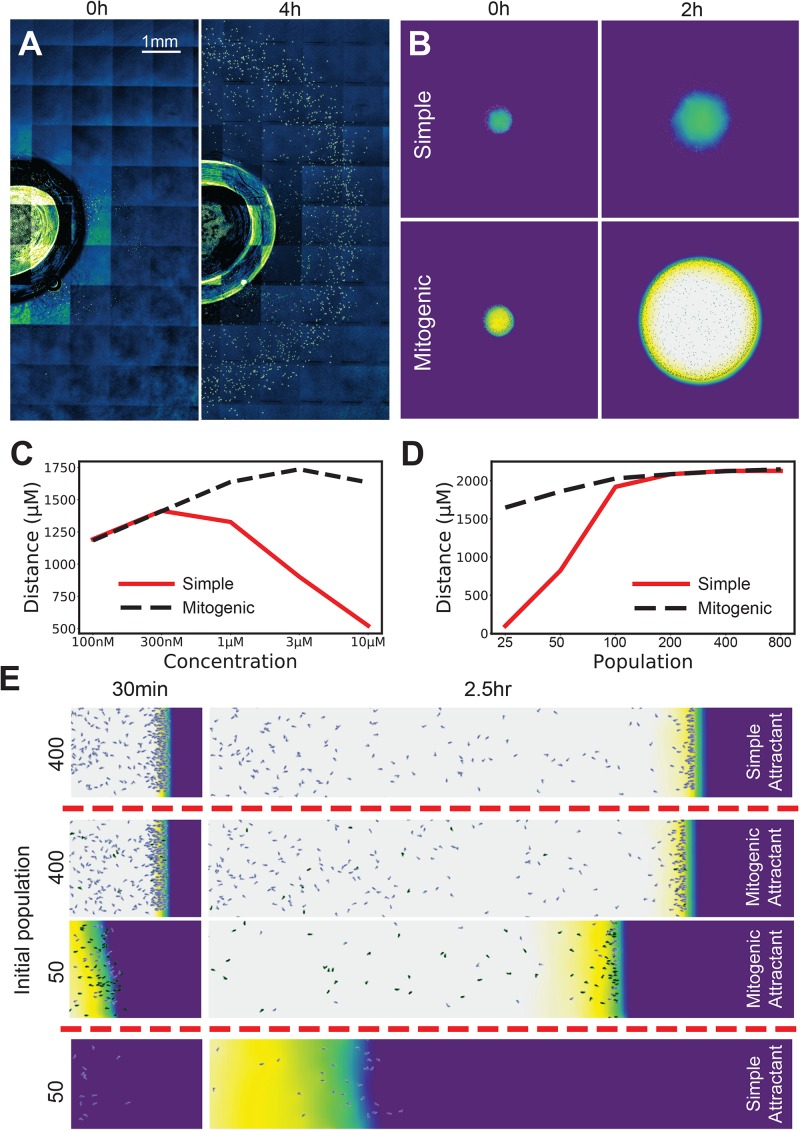
Mitogenic attractants improve the robustness of self-generated gradients to concentration, population and topological effects. **(A)** Ring-shaped wave of cells emerging from a 1 mm radius well in a 10 μM background of folate at 0 h (LHS) and 4 h (RHS). **(B)** Simulations of a small spot of cells allowed to freely migrate in an attractant background. With a simple (non-mitogenic) attractant, cells progress for a moderate distance, then stall as the circumference of the wave expands to a point where the influx of attractant requires more cells to degrade it than are present. In contrast, a mitogenic attractant (i.e., an attractant that stimulates population growth at high concentrations) causes population expansion exactly where it is needed, making the migration far more robust to the drawback of the initially small founder population. **(C)** Radial distance traveled by the leading cells across a range of concentrations for simple and mitogenic attractants. **(D)** Distance traveled in a 10 μM background by a linear wave of cells for simple (red) and mitogenic (black, dashed) attractants (compare with 2D- the red line is the same data in both panels). **(E)** Initial populations of 400 and 50 cells with a mitogenic attractant, compared against their counterparts with a simple attractant from Figure 2A.

The loss of robustness as the number of cells decreases becomes particularly important in 2D and 3D assays. Most laboratory assays for chemotaxis [for example, Zigmond/Dunn/Insall chambers (Zigmond and Hirsch, 1973; Zicha et al., 1991; Muinonen-Martin et al., 2010), transwell assays (Zigmond and Hirsch, 1973) and single-track under-agarose assays as shown in Figure 2B] are inherently one-dimensional; a group of cells follow a path of essentially constant width. In 2D assays such as circular invasion (Yu and Machesky, 2012; Susanto et al., 2016) or one-spot assays (Figure 3A), the migration front of the chemotactic cells constantly increases in circumference, so low cell density will inevitably be a problem after cells have migrated a significant distance. Three-dimensional assays are even more profoundly affected because the front expands more rapidly. This means that robust chemotaxis in *in vivo* conditions requires a mechanism that allows cells to compensate.

### Robustness Is Reinforced if the Attractant Is Also a Mitogen

We have observed that chemoattractants are frequently also mitogens. Examples include the lipid mediators LPA and sphingosine 1-phosphate (Goetzl and An, 1998), endothelin 3 during melanoblast development (Horikawa et al., 1995), and chemotactic growth factors such as FGF and VEGF (Chicoine and Silbergeld, 1997). This suggests a solution to the problem of 2D/3D chemotaxis. As the migrating front expands, diminishing cell density causes chemotaxis to fail (see Figure 3B, simple attractant). The reason is the same as in our earlier low-population experiments – the attractant flux can overwhelm attractant degradation by the rarefied cells, thus saturating their receptors and abolishing guidance cues. If the attractant is also a mitogen, saturating attractant will drive a maximal rate of cell division whenever there are too few cells (Figure 3B, mitogenic attractant). At lower concentrations this is not as quickly required, and so no effect is observed (Figure 3C). Thus, mitogenic chemoattractants add substantially to the range of concentrations at which cells can migrate in a circular assay (Figure 3C, re vs black lines). A mitogenic effect also compensates for saturation related delays in one-dimensional assays (Figure 3D), allowing an initially small number of cells to migrate as effectively as a large number (Figure 3E).

We conclude that chemoattractants that are also mitogens add great robustness to conditions where the front increases in size over time or when the initial number of cells is small, for example, during neural crest migration in metazoan development.

### Induced Degradation Improves the Range of Sensitivity

The small-population assay (also known as the two-spot assay) uses a very small number of cells, in the hope that there will not be enough to break down significant amounts of attractant (Konijn, 1970). In this assay, a small (∼500 nl) spot of cells is placed on agar about 500 μm from a similar spot of attractant. This assay gives responses over an exceedingly broad dynamic range – we have routinely found cells sensitive to stimuli whose concentration ranges of over six or more orders of magnitude. This is hard to accommodate with a model in which the chemotactic gradient is imposed and not degraded, where (as discussed earlier) attractants must be presented within about an order of magnitude of the receptor’s K*_*d*_*. We therefore examined the role of attractant degradation.

**FIGURE 4:**
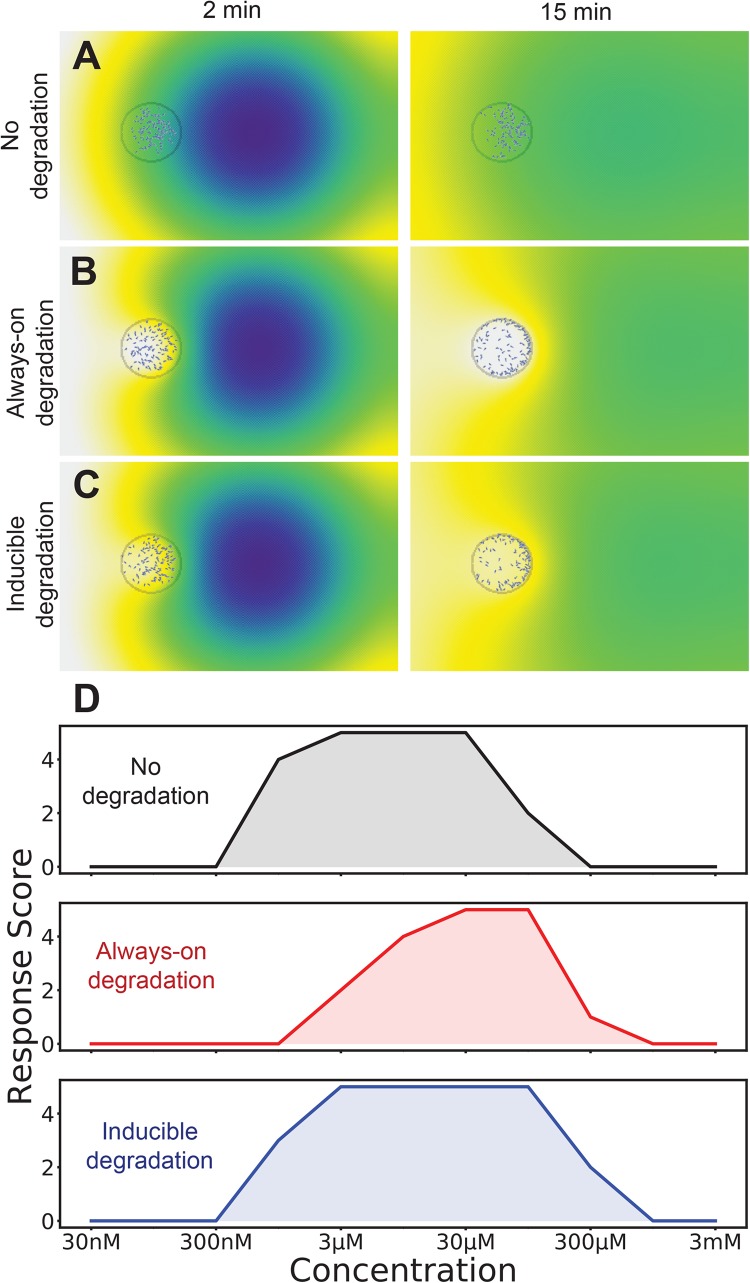
The best of both worlds: induction of attractant degradation retains the high-concentration responsiveness of self generated gradients and low concentration sensitivity of non-degradable systems. **(A–C)** Simulation of two-spot assay at 10 μM without degradation **(A)**, with always-on degradation **(B)**, and with degradation induced as an ultrasensitive function of receptor occupancy **(C)**. A simulation is scored as positive if twice as many cells are in the right-half of the cell drop as the left half. **(D)** Number of tests that score positive for each degradation logic across a range of attractants (out of a total of five).

Figure 4 shows the modeled behavior of *Dictyostelium* cells that do not (Figure 4A) and do (Figure 4B) break down the attractant cyclic AMP. The resulting chemoattractant profile is very different under these two conditions. Without degradation, there is a gentle positive (left to right) gradient. In contrast, the predicted profile of the degraded attractant is almost radial, though it still climbs more steeply toward the initial attractant location. The distribution of the cells is strikingly different too – with no degradation the cells mostly stay in the middle of the drop, biased toward the front half. With degradation, the cells cluster all around the edge of the drop, still favoring the front. The real behavior of cells in two-spot assays strongly suggests that even these tiny populations can degrade the influx of cAMP (see for example, Insall et al., 1996). It is worth noting that this assay traditionally scores a drop of cells as positive or negative, with no more subtle quantification. As both of these examples have twice the number of cells in the front half of the spot, both score identically in spite of their differences.

We hypothesized that the robustness of the responses would be increased if the chemoattractant-degrading enzyme were directly induced by high chemoattractant levels. This has been measured for both cAMP and the alternative chemoattractant, folate (Bernstein et al., 1981), in *Dictyostelium*. We introduced an induction mechanism for attractant degradation, coupling the average receptor occupancy of a cell over the last minute to its maximal degradation rate. We gave this a hill coefficient of two, so that there would be a switch-like response as concentration increased. As shown in Figure 4C this does not substantially alter the predicted distribution of cells.

The behavior of cells with and without degradation is qualitatively very different, however if we simply employ the binary score system their respective dynamic ranges are similar, though at slightly different absolute levels. Without degradation, modeled cells respond to spots of between 1 and 100 μM, whereas degrading cells respond to 3 and 300 μM spots (the concentration that reaches the cell is of course smaller). However an inducible enzyme extended the range, allowing small populations to respond to spots between 1 and 300 μM (Figure 4D). Thus direct induction by attractants of the enzymes that metabolize them increases robustness of chemotaxis.

It should be noted that the dynamic range of the model is still not as good as is seen for real cells. There could be several reasons for this. First, this model explicitly models a 2D surface containing the attractant – in reality, the agar on which the spots sit *in vitro* is much thicker than the distance between the cell and attractant spots – losses in 3D for such a small drop may lead to an apparently higher dynamic range. Second, the model, which is based on published parameters, may understate the sensitivity of cells. Third, the time delay described in Figures 1D,E also adds robustness; cells are scored positive if they are in the front of the drop, whether they move early or late in the assay. Finally, there may be additional mechanisms making chemotaxis yet more robust to different attractant concentrations.

## Discussion

The first conclusion from this work is that self-generated gradients work in an entirely different way to imposed gradients. When cells break down attractants, they can create gradients that are tailored to their own optima for responsiveness. This is true whether the gradients are 100% self-generated – made newly from a homogeneous environment – or reshaped by attractant breakdown from a higher-concentration and less sharply focused imposed gradient. In both cases, the fact that the cells can alter the gradient as they respond to it allows great robustness; if the attractant concentrations are not appropriate, the cells can reduce them, in whatever numbers make the most legible gradient, taking whatever length of time is ideal, and altering their own behavior by inducing enzymes or cell division as appropriate.

In this work we have primarily focused on extracellular, membrane-bound enzymes as the drivers of attractant breakdown. Other mechanisms are possible, and indeed have been found *in vivo* – for example, endocytosis of signaling receptor-ligand complexes (Sorkin, 2001), and scavenger receptors (Ford et al., 2014), which are related to signaling receptors but evolved specifically to remove ligand. Enzymes have a faster substrate turnover than endocytosed receptors, so they are suitable for particularly abundant or diffusive ligands. The chemoattractants cAMP and LPA are in this category. Other attractants, such as growth factors and chemokines, are larger and act at lower concentrations, so a gradient can efficiently be set up by receptors. One-dimensional migrations – the zebrafish lateral line primordium, for example (Boldajipour et al., 2008), are particularly well suited to receptor-based mechanisms because the rate at which attractants can arrive is relatively slow. There is no difference in process between receptor- and enzyme-mediated breakdown, just different affinities and reaction rates; probably in many cases both are used simultaneously.

The situations described in Figures 3, 4 – attractants as mitogens, and attractants inducing the enzymes that will break them down – have been observed in multiple instances *in vivo* (FGF, LPA and endothelin-3 are mitogens, and the *Dictyostelium* chemoattractants cAMP and folate each induce their own breakdown enzymes). Given the improvements in robustness that are available, we expect both scenarios will be seen more often. We predict many embryonic chemoattractants will also turn out to be mitogens, because embryos face exactly the problems we have addressed – small numbers of precursor cells, migrating in three dimensions to a target that is large and growing. We also predict that chemotactic cells that must respond to relatively few chemoattractants (CCL2 for macrophages, or CCL19 for T-cells) will often respond to high basal levels by inducing attractant-breaking enzymes.

We believe the large majority of chemotaxis *in vivo* involves at least some self-generation. The limitations on non-degradable, imposed gradients both concerning concentration range and maximal distance mean that it cannot serve as the primary mechanism of migration in many circumstances. There are technical challenges with observing self-generated gradients *in vivo*, though. Mass spectrometry has been used to confirm a predicted attractant distribution in a self-generated gradient (Tweedy et al., 2016a), but the assay is destructive and does not allow dynamic observation. In contrast, receptor turnover was used to infer concentrations dynamically in the zebrafish lateral line, but this does not allow direct observation of the true attractant concentrations (Donà et al., 2013; Venkiteswaran et al., 2013). As observational and microscopy techniques improve, we expect to find self-generated gradients, and the robustness-enhancing mechanisms described here, in an increasing range of physiological systems; they may even be seen as diagnostic of chemotaxis when found in the future.

## Methods

### Simulations

Simulations couple individual, moving agents to an environmental attractant in the same manner as (Tweedy et al., 2016a), with modifications accounting for mitosis and induction of degradation. Briefly, we use a background grid containing attractant concentration information. The grid simulates diffusion in the environment with the semi-implicit DuFort Frankel method. In simulations of imposed, non-degradable attractants, gradients form between special parts of the grid that hold their concentration at its initial level, effectively creating infinite sinks and reservoirs between which gradients can form. We do this, rather than simply assuming a linear gradient at all times, so that the brief periods in which the gradient is exponential can contribute to the resulting behavior of cells (for example, they may cause cells to commit to an initial direction more effectively). In simulations of self-generated gradients, cells degrade attractant at a rate *r* determined by Michaelis–Menten kinetics, i.e.,

$$r=V_{m\,a\,x}\,\frac{c}{c+K_{m}}$$

Attractant is removed evenly from all grid points overlapped by the cell, with the cell assumed to overlap all grid-points within 6 μm of its centroid.

Cells are individual agents moving with a persistent, locally biased random walk. An initial new direction is drawn from a wrapped normal distribution. This represents the persistent random element of their behavior, and causes them to favor their current direction of motion in the absence of other stimuli (Insall, 2013). A second, bias-driven movement is then calculated from the concentration gradient direction of only those grid points that the cell overlaps. This is multiplied by a bias strength parameter, which influences how much cells reorient in the presence of new information, and the two vectors are added together to give a final direction of motion. The cell then moves with constant speed in this new direction.

In Figure 3, some simulations allow the attractant to act as a mitogen. In these cases, cells maintain a running average occupancy over the last minute. The expected time to the next mitosis interpolates between infinite at a minimum average occupancy of 0.35 to 1 min at an occupancy of 1. Following mitosis, the running average occupancy for that cell is set to zero to introduce a (brief) refractory period.

In Figure 4, we introduce inducibility to the degradation mechanics of each cell. When attractant degradation is inducible, the rate of degradation is multiplied by an additional factor tracking the currently induced level:

$$r=V_{m\,a\,x}\,\frac{c}{c+K_{m}}\,\left(v_{b}+\left(1-v_{b}\right)\,\frac{y_{\mu }^{h}}{k_{i}^{h}+y_{\mu }^{h}}\right)$$

where *y*_μ_ is the running average occupancy, *v*_*b*_ is the unitless basal level of activity, *k*_*i*_ is the occupancy of half-activity and *h* is the Hill coefficient. In our case, *v*_*b*_ = 0.05, *k*_*i*_ = 0.4, and *h* = 2.

Simulations are written in Java and the source code is available on GitHub at https://github.com/ltweedy/Robustness_ABM.

### Long Range Chemotaxis Experiments

Our long range experiments are a variation on the *Dictyostelium* under agarose assay [Laevsky and Knecht (2001) Biotechniques 31:10.2144/01315rr03]. Vegetative *D. discoideum* strain NC4 are grown on bacteria/SM agar. Cells are introduced into a ∼20 mm × 4 mm well cut into 2 mm deep 0.4% agarose/LoFlo in a 50 mm glass bottomed dish (MatTek). The agarose contains a chosen concentration of folic acid as the attractant, uniformly mixed in as the agarose cools (folic acid is very unstable at high temperatures). The volume of cell suspension introduced to the well is constant throughout these experiments (a little under 160 μl), but the number of cells/ml, and so, the size of the whole inoculum, varies depending on the specific experiment.

One variation is presented, in which general appearance is compared to simulation (rather than quantified results). In Figure 3, we present the same kind of experiment, but the well is created using a 2 mm wide biopsy punch, ensuring a radial spread of cells rather than a largely linear one.

Imaging took place on a Nikon Eclipse TE2000-E using a 10× phase contrast objective, with image capture performed every 2 min by a Retiga R6 camera under the control of Micromanager.

## Data Availability Statement

The datasets generated for this study are available on request to the corresponding author.

## Author Contributions

LT conceived the work, performed simulations and lab work, analyzed the data, and edited the manuscript. RI conceived the work, and drafted and edited the manuscript.

## Conflict of Interest

The authors declare that the research was conducted in the absence of any commercial or financial relationships that could be construed as a potential conflict of interest.
